# Successful Cardiopulmonary Resuscitation in a Sevoflurane Anaesthetized Horse That Suffered Cardiac Arrest at Recovery

**DOI:** 10.3389/fvets.2018.00138

**Published:** 2018-06-22

**Authors:** Clara Conde Ruiz, Stéphane Junot

**Affiliations:** ^1^Service d'Anesthésie, VetAgro Sup, Université de Lyon, Marcy L'Etoile, France; ^2^Unité APCSe, Agressions Pulmonaires et Circulatoires dans le sepsis, VetAgro Sup, Université de Lyon, Marcy L'Etoile, France

**Keywords:** horse, cardiac arrest, CPR, bezold jarisch, complications, anaesthesia

## Abstract

A 17-year-old mare undergoing dental surgery suffered a cardiac arrest while being transferred from the surgical theatre to the recovery box. This complication was diagnosed early, thus allowing a prompt start to the cardiopulmonary resuscitation maneuvers. External thoracic compressions, intermittent positive pressure ventilation, and adrenaline administration were at the core of this successful resuscitation. Although it was not possible to confirm the cause of cardiac arrest in this horse, a Bezold-Jarisch reflex due to potential decrease on venous return because of postural change and drug interactions was hypothesized. Based on this report, it appears advisable to smoothly change the position of anaesthetized patient; furthermore, the administration of drugs affecting cardiovascular hemodynamics or sympatho-vagal balance to animals while changing their recumbency should be avoided.

## Introduction

A perioperative mortality rate from 0.12 to 0.9% has been reported in non-colic horses (1, 2). Despite the advances and improvements in equine anaesthesia, mortality rate remains still high (3). A third of these fatalities is due to cardiac arrest, as reported in sick and healthy horses (1). Severe debilitating diseases and anaesthetic drugs-related factors have been described as leading causes of cardiac arrest in horses (1). The occurrence of cardiac reflexes following change of hemodynamic conditions may also lead to this complication.

Peri-anaesthetic cardiac arrest, despite being uncommon, carries a poor outcome in horses (1, 2, 4), mainly because of the difficulties performing cardiopulmonary resuscitation (CPR) maneuvers in this specie. An early detection of the complication and a trained staff are key-points for the potential success of CPR. The present case aimed to describe the successful detection and treatment of a cardiac arrest occurring after a postural change during the recovery phase of anaesthesia in a horse.

## Background

Written informed consent was obtained from the owner for the publication of this case report. A 17-year-old Hanoverian mare, 550 kg, was admitted to the Equine Hospital for surgical removal of a fractured tooth (tooth #308, modified Triadan Tooth Numbering System of equine dental nomenclature). Preoperative physical examination, electrocardiography (ECG) and blood tests were unremarkable. Therefore, the American Society of Anaesthesiologists (ASA) physical status was classified as two. Due to the complexity of the surgical procedure, the mare was scheduled for tooth removal under general anaesthesia the following day.

Food was withheld for 12 h with free access to water. In the day of surgery, a 14-gauge catheter was inserted percutaneously into the left jugular vein.

Acepromazine (Calmivet®, Vetoquinol, France) 0.04 mg kg^−1^ was administered intramuscularly (IM), phenylbutazone (Phenylarthrite®, Vétoquinol, France) 2.2 mg kg^−1^ and trimethoprim-sulfadoxine (Borgal®, Virbac, France) 15 mg kg^−1^ were administered intravenously (IV) 1 h before anaesthesia. In the induction box, the patient was sedated with romifidine (Sedivet®, Boehringer Ingelheim, France) 0.04 mg kg^−1^ IV followed 5 min later by morphine (Morphine Clorhydrate Aguettant®, Aguettant, France) 0.1 mg kg^−1^ IV. Anaesthesia was induced with diazepam (Valium®, Roche, France) 0.05 mg kg^−1^ and ketamine (Imalgène1000®, Merial, France), 2.2 mg kg^−1^ IV, given in separate syringes. Endotracheal intubation was performed with a 26 mm internal diameter silicone tube and the patient hoisted to the theatre and positioned in right lateral recumbency.

Once in theatre, the horse was connected to a circle breathing system and anaesthesia was maintained with sevoflurane (Sevoflo®, Axience, France) in 100% oxygen. The inspired fraction of sevoflurane was titrated to effect to maintain an adequate depth of anaesthesia based on clinical signs (palpebral reflexes, position of the eye, absence of nystagmus). A 20-gauge cannula was placed in the left metatarsal artery for invasive blood pressure (IBP) monitoring and regular arterial blood sampling for blood gas analyses. Vital signs monitoring was performed with a multivariable monitor (Datex S/5, GE Healthcare, UK) and consisted in continuous ECG, heart rate (HR), oxygen saturation (SpO_2_), inspired and expired fraction of carbon dioxide (P${}_{{\text{E}}^{\text{′}}}$CO_2_), inhaled and end-tidal concentration of oxygen and sevoflurane and IBP. Ringer Lactate (Ringer Lactate Aguettant®, Aguettant Laboratories, France) was infused at 10 mL kg^−1^ hour^−1^. Dobutamine (Dobutamine Panpharma®, Panpharma, France) IV was administered at 2–10 μg kg^−1^ min^−1^ to effect, to maintain mean arterial pressure (MAP) above 70 mmHg. Intermittent positive pressure ventilation (IPPV) was provided using a volume cycled - pressure controlled ventilator (Stephan Respirator-GT; F. Stephan GmbH, Germany), and settings were adapted to maintain a P${}_{{\text{E}}^{\text{′}}}$CO_2_ between 4.6 and 6.0 kPa (35 and 45 mmHg). Additional analgesia was provided with lidocaine (Lurocaine®, Vétoquinol SA) 1.5 mg kg^−1^ IV administered over a 20-min period, followed by a constant rate infusion (CRI) (50 μg kg^−1^ min^−1^).

Trepanation for removal of the tooth #308 was performed, followed by a cleaning and a curettage of the dentary alveoli into which a silicone temporary prosthesis was placed.

Anaesthesia lasted 130 min, with a surgery duration of 75 min. No surgical complications were reported. Except for a period of 10 min after induction in which MAP values were around 60 mmHg, no other events or abnormalities on the ECG, HR or MAP were observed during anaesthesia. Ninety minutes after induction, morphine 0.1 mg kg^−1^ IM was administered, and the lidocaine CRI was stopped 20 min before the end of anaesthesia. Ten minutes later, the horse was weaned from the ventilator. At the end of the surgical procedure, 5 min before the end of anaesthesia, the intra-arterial catheter was removed, fluids were stopped but the IV catheter was kept for recovery. The vaporizer and oxygen were switched off; the horse was disconnected from the monitoring devices and from the breathing system but remained orotracheally intubated. While the horse was attached to a hoist and positioned on dorsal recumbency, a romifidine 0.02 mg kg^−1^ IV bolus was given, and the horse moved thereafter to the recovery box. Once there, the horse was positioned on right lateral recumbency on a padded floor, with the anaesthetist at its head to check vital signs and avoid premature attempts of rising. The time from the end of anaesthesia to this point was approximately 2 min. Despite the horse was breathing spontaneously before its transfer, apnoea was noticed when positioned in the recovery box. At physical examination, pulse was absent, mucous membranes were greyish, and pupils mydriatic. Cardiac auscultation confirmed the absence of cardiac beats. The time was noted, and thoracic compressions were immediately started by an operator jumping with his knees on the mare's thorax. Three persons (weighting 60, 80, and > 90 kg, respectively) rotated every 2 min to perform the external massage. The third heavier operator performed massage by rhythmically and energetically sitting on the horse's thorax. Meanwhile, 6 mg of adrenaline (Adrenaline Aguettant®, Aguettant, France) was administered IV, followed by 5 mL of heparinised saline. Mechanical ventilation was provided with a demand valve at a rate of 10 breaths min^−1^, with 100 % oxygen and Ringer Lactate was administered at 10 mL kg^−1^ hour^−1^. Vital parameters were continuously monitored by mandibular pulse palpation, eye reflexes evaluation. While the third operator was performing the external massage, mandibular pulse was detectable and synchronous to the thoracic compressions. A multiparameter monitor was then connected for ECG, HR, and non-invasive blood pressure measurements, with a cuff on the left metacarpal bone. However, due to the movements on the patient, monitoring assessment was difficult. Five minutes after the start of CPR, the anaesthetist detected a stronger mandibular pulse, asynchronous to the external massage. Thoracic compressions were stopped, and the anaesthetist confirmed the presence of normal QRS complexes on the ECG, whereas the horse was still apnoeic. Mechanical ventilation was continued with the demand valve with a respiratory rate of 6 breaths min^−1^. The mare was initially tachycardic (HR of 60 beats min^−1^), with a sinus rhythm, and MAP of 80 mmHg. Within the next 10 min, HR decreased to 37 beats min^−1^ and MAP dropped to 50 mmHg with a poor pulse quality. Dobutamine CRI was thus administered to effect at 0.5 to 2 μg kg^−1^min^−1^ IV for 5 min, until MAP reached 70 mmHg, and stopped thereafter. Ten minutes after the return of spontaneous circulation, spontaneous breathing reappeared. Afterwards, IPPV was stopped but oxygen supplementation was continued using a flow-by method, with an oxygen supply tubing positioned in the endotracheal tube and an oxygen flow set at 12 L min^−1^. At this time, pupillary reflex was present, but not palpebral reflex. Capillary refill time was less than 2 sec and SpO_2_ 100%, but mucous membranes remained pale pink and sweating was present. An arterial blood gas analysis was carried out and revealed a non-compensated respiratory acidosis (pH 7.3; arterial pressure of carbon dioxide (PaCO_2_) 67 mmHg; bicarbonate, 29 mmol L^−1^; anion gap 14 mmol L^−1^, base excess 0 mmol L^−1^). Palpebral reflex and nystagmus were noticed 15 min after the return to spontaneous circulation. Ten minutes later, reflexes became stronger and the horse started presenting some movements, which were controlled by two operators at the head to avoid premature standing. The endotracheal tube was secured to the horse's mouth and all equipment was removed from the box to prepare for the recovery, which was assisted with ropes. The mare stood up at the first attempt 1 h after the start of CPR, and remained quiet thereafter, although trembling. The patient was then extubated and kept in the recovery box for close observation. Two hours later, the mare was transferred to the hospitalization box and received phenylbutazone 4.4 mg kg^−1^ IV and omeprazole (Gastrogard, Merial, France) 2.2 mg kg^−1^, orally. Venous blood sample analysis revealed a lactate into the normal range (1.3 mmol L^−1^) and mild increased creatinine kinase (751 IU L^−1^). Neurological examination was normal thereafter: the horse was alert with normal pupillary reflexes, no apparent blindness, deafness or ataxia.

The postoperative period was uneventful. Two days after anaesthesia, a cardiac ultrasound was performed, which did not reveal any abnormality. Due to the favorable outcome, the patient was discharged from the hospital 1 week later.

## Discussion

This case reports the successful resuscitation of a 17-year-old, 550 kg mare undergoing tooth removal under general anaesthesia that suffered cardiac arrest while transferred to the recovery box.

Cardiac arrest is a complication of equine anaesthesia that has been poorly studied. Risk factors regarding perioperative mortality include an increased ASA physical status, age, surgery type, prolonged duration of anaesthesia and emergency procedure (1). In the present case, it was debatable if a 17 years old horse could be considered as geriatric. If so, the patient could have presented a decreased ability to respond to circulatory changes or stress and, therefore, at a higher risk to anaesthetic complications (5). However, despite its age, the mare was considered overall healthy with no detected systemic abnormalities and no exercise intolerance; vital signs remained remarkably stable during anaesthesia. Therefore, in this case, the occurrence of the cardiac arrest was difficult to predict but, fortunately, its early detection allowed the prompt start of CPR maneuvers.

Cardiopulmonary resuscitation aims to restore spontaneous circulation and breathing and avoid irreversible hypoxic damages to organs. The probability of success for CPR in adult horses is considered as poor (6). The size of the animal and the physical effort required to provide cardiac massage render this procedure complicated to perform. In addition, the lack of advanced monitoring during recovery may delay the detection of cardiac arrest and worsen the outcome.

Although cardiac arrest involves one third of equine perioperative mortality (1), there is a lack in literature regarding its occurrence and treatment in adult horses. Successful CPR after direct cardiac massage in a pony and a horse was reported by De Moor et al. (7), however, both animals died in post resuscitation period. Hubbell et al. (8) evaluated the effects of thoracic compression rate on cardiac output in horses with induced cardiac arrest. They reported that thoracic compressions at a rate of 80 compressions min^−1^ allowed a better cardiac output, in comparison with lower rates. Moreover, cardiac output was higher when the operator was heavy. In this last study, horses were on right lateral recumbency and the operator delivered a blow to the chest wall immediately posterior to the left elbow with his knees. In the present case, thoracic compressions were performed in a similar way. Despite the aim was to perform 80 compressions min^−1^, this rate seemed very difficult to achieve in practice and the rate observed in our case was probably closer to 40 to 60 compressions min^−1^. The third operator was the heaviest and most experienced surgeon; instead of compressing the horse's thorax with his knees, he used his whole core body by sitting on it. A better pulse quality was subjectively achieved with this way of performing the external massage, but it could also have been attributed to the heavier weight of the operator.

In addition to these physical maneuvers, adrenaline was administered to the horse. This drug is recommended for asystole in horses (6) and small animals (9). Adrenaline is a synthetic catecholamine with strong α_1_- and β_1_-, and moderate β_2_- adrenergic receptor activity which produces vasoconstriction and an increase in HR and contractility (10). In the present case, a single low dose (0.01 mg kg^−1^) IV was used. Despite it was difficult to evaluate which part of the resuscitation maneuvers contributed to the return of spontaneous circulation, it was probably the combination of both, thoracic compressions and adrenaline that contributed to the successful CPR.

In addition to the external massage, the basic life support consists in ventilation. As previously described (8, 11), IPPV using a demand valve was performed early during CPR, at a rate of 6–10 breaths min^−1^. Even though we cannot be certain of the minute ventilation provided, it probably allowed sufficient oxygenation of the animal, as no clinical signs of hypoxic brain damage were noticed thereafter.

In this case, the lack of close monitoring during the horse transfer made difficult to determine the precise moment and the real cause of the cardiac arrest. It was unlikely to be related to the animal health status, although individual idiosyncrasy could not be excluded, but may have been due to the surgical procedure, the occurrence of cardiovascular reflex or drug effect. Firstly, there was a potential risk of embolism associated with bleeding. However, no bleeding was noticed, nor was any sudden variation of P${}_{{\text{E}}^{\text{′}}}$CO_2_ during the procedure. Second, a cardiac reflex following a postural change could be considered in this case. Severe cardiovascular depression in similar circumstances has been reported in a dog (12) and in humans (13, 14). In this case, the horse experienced a rapid postural change for its transfer to the recovery box. This could have produced a compression of the caudal vena cava by abdominal viscera, leading to a decreased venous return for a few seconds, the so-called Bezold- Jarisch reflex (BJR). The BJR is a complex neuro-cardiogenic reflex mediated by ventricular receptors sensitive to chemical and / or mechanical stimuli in response to a decreased ventricular filling (15), that is not necessarily related to a hypovolaemic state. The afferent pathway is vagally mediated and terminate at the nucleus tractus solitary in the central nervous system (16). The efferent response produces an increased parasympathetic tone, leading to bradycardia, vasodilation and apnoea (17). In addition to a postural change, an interaction of a variety of anaesthetic drugs may trigger this reflex (18).

Therefore, the drugs used in the present case may also have participated to the observed complication. After its use for premedication without noticeable adverse effect, romifidine 0.02 mg kg^−1^ IV was administered at the time the horse was attached to the hoist. In our practice, this drug is routinely administered at the end of anaesthesia to improve the quality of recovery of horses (19). As an α_2_-agonist, romifidine produces an initial increase in blood pressure with a subsequent bradycardia; second degree atrioventricular blocks and a decrease in stroke volume are commonly reported (20). Despite cardiovascular effects of romifidine may be dose dependent, to the authors' knowledge there are no studies that evaluated the cardiovascular effect of low dose of romifidine in anaesthetized horses. Even though it seems unlikely that romifidine alone would have been responsible for this complication, we cannot exclude a role in the development of the cardiac arrest.

Among the other drugs administered, acepromazine, a phenothiazine derivate and α_1_-adrenoreceptor antagonist, was used in the premedication. In addition to hypotension (21, 22), phenothiazine derivates have been associated in humans to a decrease in myocardial contractility (23) however, this has not been reported in horses. Conversely, it has been associated to a protective cardiac effect and a decreased anaesthetic mortality (1). Even though it may have a prolonged effect on blood pressure, it was unlikely to have promoted the cardiac arrest as blood pressure was overall well maintained during anaesthesia in this case. Sevoflurane was used for maintenance of anaesthesia. In healthy horses, it produces a dose dependent decrease in MAP, cardiac index and systemic vascular resistance (SVR) (24). This decrease in SVR could have contributed to worsen the potential decrease in venous return hypothesized in this case. Similarly, lidocaine, a local anaesthetic commonly used in equine anaesthesia, may produce bradycardia and hypotension at high doses (25) and may have participated to the altered cardiovascular response to the postural change, even though it was stopped 20 min before the end of anaesthesia. Dobutamine, a β_1_- adrenoreceptor agonist with inotropic properties, commonly used for treating hypotension in anaesthetized horses, was also associated with the occurrence of BJR in a dog (26). As it was sparely used in this case and particularly not at the time of recovery, it was unlikely that it had any influence on the onset of the reported complication. Finally, fatal reactions to trimethoprim-sulfadoxine have been reported in anaesthetized horses (27, 28). However, in this case, trimethoprim-sulfadoxine was administered 1 h before anaesthesia, which made it unlikely as a cause of the adverse event observed.

Based on the chronology of events, we hypothesized that the postural change combined with the cardiovascular effects of the different anaesthetic drugs used triggered the occurrence of a cardiac arrest, due to a possible BJR. However, the absence of monitoring during the transfer of the animal rendered difficult to confirm this assumption. The early detection of the complication and the presence of trained staff allowed a successful outcome. The present case also underlined that the equine patient is at high risk during the recovery period not only regarding the risk of trauma but also regarding the risk of cardiovascular and respiratory instability.

## Concluding remarks

The early detection of cardiac arrest and start of CPR maneuvers permitted the successful resuscitation of a 17-year-old mare that suffered cardiac arrest during her transfer to the recovery box.

In the light of this report, a continuous palpation of the peripheral pulse should be performed during the horse transfer to the recovery box. It seems advisable to administer drugs with a potential depressive cardiovascular effect in a time frame that does not overlap with postural changes.

## Author contributions

CC and SJ participated in the development of the case and wrote or contributed to the writing of the manuscript.

### Conflict of interest statement

The authors declare that the research was conducted in the absence of any commercial or financial relationships that could be construed as a potential conflict of interest.
